# Commentary on Camell et al., Aging Induces Nlrp3 Inflammasome Dependent Adipose B Cell Expansion to Impair Metabolic Homeostasis

**DOI:** 10.20900/immunometab20200011

**Published:** 2020-02-18

**Authors:** Sara SantaCruz-Calvo, Lucia SantaCruz-Calvo, Barbara S. Nikolajczyk

**Affiliations:** 1 Department of Pharmacology and Nutritional Sciences, Barnstable Brown Diabetes and Obesity Research Center, University of Kentucky, Lexington, KY 40536, USA; 2 Department of Chemistry and Food Technology, Technical University of Madrid, Madrid 28040, Spain

**Keywords:** obesity, aging, inflammation, B cells, adipose tissue, FALC

## Abstract

The burden of aging and obesity is urging extended investigation into the molecular mechanisms that underlie chronic adipose tissue inflammation. B cell-targeted therapies are emerging as novel tools to modulate the immune system and thereby mitigate aging and obesity-related metabolic complications.

The world population is drastically aging: reports from the National Institute on Aging and U.S Census Bureau estimated an increase in the percentage of 65+ population from 8.5% in 2015 to 17% by 2050 [1]. The parallel obesity epidemic has reached alarming proportions with 2016–2018 CDC reports stating a prevalence of 40.1% in the 60+ population [2]. Age-associated increases in adiposity and adipose tissue (AT) redistribution, along with accelerated aging of AT in obesity associate with a wide range of health complications such as type 2 diabetes (T2D), cardiovascular disease and non-alcoholic fatty liver disease. This combination of demographic and health concerns translates into an urgent need for the scientific community to investigate molecular mechanisms underlying AT inflammation to repurpose or develop new immunotherapies to overcome the burdens of obesity and aging. Camell and colleague’s study is a perfect example of this strategy as they unveil the clinical advantages of depleting a unique aged but non-senescent visceral adipose tissue (VAT)-resident B cells population, termed aged adipose B cells (AAB), to improve whole-body homeostasis and thermogenesis in older mice [3].

AT is the largest endocrine and immunological organ in obesity, so it regulates not only lipid metabolism but also glucose homeostasis, insulin sensitivity and systemic inflammation. Understanding AT is therefore critical for understanding aging and obesity, both of which are characterized by sustained low-grade inflammation. Abnormalities in the AT, especially the VAT, lead to the expansion of AT-resident immune cells and infiltration by circulating immune cells. Over the past decade, studies on changes in the AT immune system, especially during obesity [4] and to a lesser extent, aging [5], has enormously increased. Although the majority of these studies focus on topics related to the polarization of AT-macrophages (ATM) from anti-inflammatory (M2) to pro-inflammatory (M1), as mediated by IFNγ secretion from AT-T cells (ATT) [6], less abundant but equally strong publications show direct impacts of lymphocytes, predominantly T cells, on AT inflammation and thus AT function and metabolic health in obesity and aging. The pathogenic role of B cells as antigen presenting cells and antibody producers during obesity and aging has been less investigated. Exceptions include diet-induced obesity experiments, which demonstrated that obese B cell-null mice showed improved insulin sensitivity due to the absence of pathogenic IgG and reduced antigen presentation to pro-inflammatory ATT and ATM [7], as well as reduced AT and circulating leptin levels [8]. Studies in obese subjects showed increased IgG production by subcutaneous adipose tissue (SAT) B cells [9], consistent with the demonstration of pathogenic IgG in obese mice. Our related work showed that human B cells are essential for promoting Th17 inflammation only in T2D, whereas myeloid cells promote IL-17 production in a disease-independent manner [8]. These studies highlight obesity-mediated derangement in generally anti-inflammatory actions of B cells in AT from young and/or lean individuals, as also evidenced by work showing that B-1a cells and/or regulatory B cells (i.e., Bregs) produce the anti-inflammatory cytokine IL-10 to rescue obese mice from insulin resistance [10]. Together these data indicate B cells are potential targets for ameliorating obesity-induced inflammation, and raise the possibility of key roles in the pathogenic VAT in aging.

Elegant work from Camell and colleagues showed that aged mice expand AAB only in VAT niches, but not in other fat pads or spleen, and that they differ from previously identified splenic aged B cells (ABC) [11,12]. These VAT B cells niches are housed in so-called fat-associated lymphoid clusters (FALCS), that are induced during inflammation and are highly vascularized to support immune cell trafficking [13]. The authors used whole-mount staining of VAT-FALCS as a tool to provide three-dimensional information about the distribution of immune cells therein. This method generated sophisticated images of aged VAT-FALCS and found that AAB were in close contact with ATM, and that AAB expansion was supported not only by recruitment from lymphatic vessels but also by AAB replication. By depleting AAB, the authors improved insulin sensitivity (but not lipolysis) and reduced T regulatory cells (Treg) frequency and PD1 expression in aged mice. These data raise the possibility that AAB targeting could be exploited to arrest or reverse metabolic declines that associate with age, perhaps as an approach to extend healthspan [14]. The authors also showed that the activation of the NLRP3 inflammasome, a multiprotein complex responsible for triggering the protease activity that activates both pro-IL-1β and pro-IL-18 to regulate VAT inflammation and remodeling [15], was essential to increase AAB number, FALCS number and lipolysis by upregulating IL-18 and the IL-1β/IL-1βR axis through post-transcriptional mechanisms. These findings extend the Dixit lab’s previous work on the NLRP3 inflammasome, in which nerve-associated aged-ATM induce lipolysis resistance in a NLRP3-dependent fashion [16].

Mechanisms that promote AAB suggest further similarities with AT-B cells in obesity that hinge on critical metabolic regulators like leptin, an adipocyte cytokine that regulates appetite and insulin resistance [17,18]. Leptin levels increase in both obesity [19] and aging (75–99 years) [20], most likely due to the development of leptin resistance in both environments [21,22]. B cells express the long isoform of the class I cytokine leptin receptor, which is wired to signaling pathways that stimulate B cell proliferation and cytokine secretion [23] and can positively regulate B cell activation in people over 65 years of age [24]. Taken together, these data raise speculation that leptin may modulate AAB replication in FALCS (Figure 1), and control B cell number/function in obesity. Additional circumstantial evidence that leptin regulates AAB includes the demonstration that leptin is higher in females versus males [25] and that AAB expansion is higher in aged female mice. Future work aimed at pinpointing similarities/differences in B cell leptin responses will be necessary to test whether B cell physiology convergently evolves in obesity and aging. In this sense, it has been demonstrated that leptin modulates IL-18 secretion in LPS-stimulated monocytes during T2D-mediated inflammation through NLRP3-inflammasome and caspase 1 activation [26], suggesting that leptin could be also be involved in upregulating IL-18 secretion in AAB. Furthermore, growth hormone receptor (GHR) deficiency improves insulin sensitivity and obesity-mediated leptin resistance in humans [27], and abrogated GHR signaling in mice reduces NLRP3 activation that correlates with pro-longevity effects [28]. Taken together, these data point to leptin as an underlying mediator in reducing ABC and AAB in aged GHR-KO mice.

Although further investigations are needed, especially in humans, Camell and colleague’s discovery raises the possibility of using AAB depletion as immunotherapy tool aimed to improve age-associated metabolic complications, such as insulin resistance and lipolysis, with the ultimate goal of extending healthspan.

## Figures and Tables

**Figure 1.**
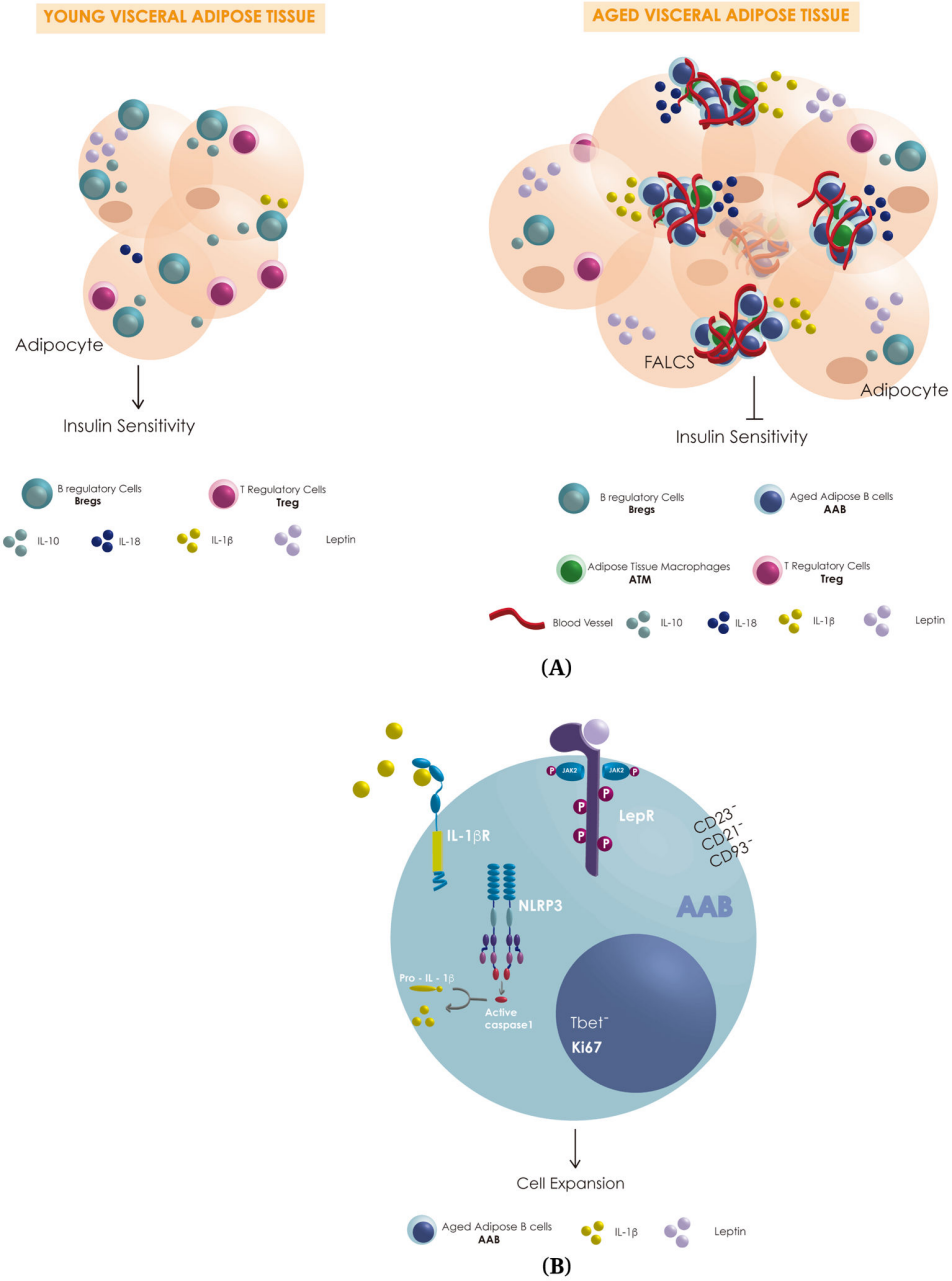
(**A**) Schematic representation of possible differences in VAT-resident immune cells (blue: AAB, green: macrophages, pink: Treg, teal: Breg; couls also be B1-a), pro-inflammatory cytokines (yellow: IL-1β, dark blue IL-18), anti-inflammatory cytokine (teal: IL-10) and leptin (purple) levels secreted by the adipocytes in young and aged-VAT-FALCS. (**B**) Schematic representation of some of the key players involved in the expansion of AAB in aged-VAT-FALCS showing the activation of the NLP3 inflammasome, caspase 1, IL-1β receptor (IL-1βR) and the possible activation of leptin receptor (LepR).
